# The Association between TNF-α, IL-10 Gene Polymorphisms and Primary Sjögren’s Syndrome: A Meta-Analysis and Systemic Review

**DOI:** 10.1371/journal.pone.0063401

**Published:** 2013-05-21

**Authors:** Baodong Qin, Jiaqi Wang, Yan Liang, Zaixing Yang, Renqian Zhong

**Affiliations:** 1 Department of Laboratory Diagnostics, Changzheng Hospital, Second Military Medical University, Shanghai, China; 2 Department of Stomatology, Changzheng Hospital, Second Military Medical University, Shanghai, China; University of Birmingham, United Kingdom

## Abstract

**Objective:**

Previous studies have evaluated the associations of TNF-α, IL-10 gene polymorphisms and susceptibility to pSS, but the results remained controversial. To assess the associations between TNF-α-308, IL-10-1082, -819, -592 polymorphisms and pSS risk, a meta-analysis was conducted.

**Method:**

The available articles were searched in PubMed, EMBASE and MEDLINE. ORs with 95% CIs were calculated to determine the strength of associations by fixed-effects or random-effects models. The data about IL-10-1082, -819, -592 polymorphisms were analyzed in the additive, dominant and recessive modes. The associations between haplotypes of IL-10 gene and susceptibility to pSS were also assessed.

**Results:**

A total of 9 relevant studies were identified in the meta-analyses. TNF-α-308 A allele was significantly associated with pSS (OR = 1.8, 95% CI: 1.53–2.13). The IL-10 -1082 G allele and the genotype “GCC/ATA” were identified as a candidate genetic risk factor for pSS. Under the dominant model for −819 and −582, the overall ORs suggested that individuals with genotype (CC+TC) or (CC+AC) may have a 59% increased risk of pSS in Caucasians population (OR = 1.59, 95% CI:1.09–1.23). Besides, the genotype “ATA/ATA” may be a protective factor against the development of pSS in Caucasians (OR = 0.40, 95% CI:0.19–0.84).

**Conclusion:**

The meta-analysis demonstrated TNF-α-308 A, IL-10-1082 G allele were significantly associated with pSS susceptibility, supporting these alleles were predisposing factors for pSS. In Caucasian population, the genotype “ATA/ATA” may be a protective factors.

## Introduction

Primary Sjögren’s syndrome (pSS) is a prototype autoimmune disease characterized by lymphocytic infiltration of exocrine tissues accompanied with a significant loss of secretory function [1]. And it primarily affects women over 40 years of age with female preponderance [2], [3]. Although the etiologic and pathogenetic mechanisms of pSS are still not fully clear, the combination of a susceptible genetic background and environmental factors has been considered to be associated with the initiation and promotion of this complex disorder.

Prevalence rates of autoimmune disorder observed in the family members of pSS patients was 30% to 35%, thus, supporting that genetic factors played a significant role in the development of the disorder [4]. Except for HLA-DRB1*03 and HLA-DQB1*02 of susceptibility genes, several associations of non-HLA factors with susceptibility to pSS have been identified [5], such as TNF-α, IL-10. As in previous studies, elevated levels of TNF-α (Tumor Necrosis Factor-α), IL-10 (Interleukin-10) were found in the peripheral blood or tissue of pSS patients, which may be related to gene polymorphisms [6], [7].

IL-10 is a multifunctional cytokine, defined as a potential trigger of secretion of autoantibodies or immunoglobulins from B lymphocytes. The IL-10 gene is located in chromosome 1, and several polymorphisms have been identified in the previous study [8]. The most widely studied of IL-10 promoter polymorphisms associated with pSS are the three polymorphisms within the proximal 1.3 kb (−1085 G/A, −819 C/T and −592 C/G). These single base-pair substitutions would mainly form three different haplotypes, GCC, ACC, and ATA. The ability to produce IL-10 may vary in accordance with the different genetic composition of the IL-10 locus [9]. Besides, as a powerful pro-inflammatory cytokine, it has been established that increased expression of TNF-αcould promote and sustain autoimmunity [10]. Several polymorphisms, such as −308 and −238, have been identified within TNF-α gene located in chromosome 6. It has also been reported that the polymorphism at the −308 of TNF-αgene could contribute to higher level of TNF-α [11].However, the associations between those polymorphisms, haplotypes and susceptibility to pSS remain unclear, the results about the role of IL-10 or TNF-αgene polymorphisms in pSS patients are inconsistent and inconclusive.

Due to these conflicting results, we have therefore conducted a systematic review and Meta- analysis of available literatures. The aim of the present study was to examine whether the polymorphisms in the promoter region of TNF-α, IL-10 were associated with susceptibility to pSS.

## Materials and Methods

### Data Sources and Searches

According to the reporting guidelines of Meta-analysis of Observational Studies in Epidemiology (MOOSE), a systematic review and meta-analysis was conducted [12]. For selection of studies, we carried out a systematic review of the electronic databases including PubMed, EMBASE, and MEDLINE independently by two investigators (BD.Q and JQ.W). We adopted the comprehensive search strategies including Mesh term and Keywords as follows: Primary Sjögren’s Syndrome, Interleukin-10 (IL-10) or tumor necrosis factor (TNF-α), and variant or polymorphism. The final date for inclusion was December, 2012 and no other restrictions on publication language, ethnicity, or geographic region were imposed.

### Study Selection

The articles were limited to studies on polymorphism of the IL-10, TNF-αgene and pSS. All included studies have to fulfill the following characteristics and inclusion criteria: (a) the study design must be a case-control study; (b) the diagnosis of pSS should meet the internationally accepted criteria; (c) there should be sufficient data for extraction or assessment of Odds Ratio (OR) with 95% Confidence interval (CI); (d) the paper must have been published in peer-reviewed journal as full article. Besides, articles would be excluded if they met anyone of the following criteria: (a) only one group included, reviews, case reports, mechanism studies as well as non-human studies should be excluded; (b) data on the same or overlapped population have been reported repeatedly; (c) there is no enough information or data for extraction; (d) unpublished studies should not be eligible for inclusion.

### Data Extraction and Quality Assessment

A standard reporting form was established to extract the information from each study, which included: first author’s name, country, year of publication, ethnicity, age, diagnosis criteria of pSS, typing technique, the total number of pSS patients and controls, and the frequency of genotype or allele. All data were independently and in duplicate extracted from all included studies by two investigators (BD.Q and JQ.W). The final results were compared by two authors and any disagreements were resolved by consensus with the other authors. As a standard quality criteria for meta-analysis of Single Nucleotide Polymorphisms studies was lacking, we used a modified Newcastle-Ottawa scale score system to evaluate the quality of these non-random studies [13].

### Statistical Analyses

The literature review was conducted according to PRISMA statement standards. The present study also coincide the minimum set of studies for reporting in the systematic review and meta-analysis [14]. The combined OR with its 95%CI was calculated in a fixed-effect or random-effect model to determine the strength of the associations between IL-10, TNF-α gene polymorphisms and susceptibility to pSS. The χ^2^-based Q test and I^2^ statistic were applied to judge the heterogeneity among studies. For Q test, P<0.10 was indicated to be representative of statistically significant heterogeneity, and I^2^ statistic represented the percentage of total variation contributed by a between-study variation ranged from 0% to 100% [15]. If there was no significant heterogeneity, a fixed-effect model would be performed to pool data. Otherwise, a random-effect model was used. The publication bias was assessed using funnel plots, Egger’s test and Begg’s test. To evaluate the impact of each individual study on the overall OR, sensitivity analysis was conducted using the one-study remove approach as previously reported [16]. Besides, Hardy-Weinberg Equilibrium was evaluated by using χ2 tests for the polymorphisms investigated in each study. All analyses were carried out in STATA 11.0 (StataCorp, College Station TX, USA). P value of less than 0.05 was considered significant.

## Results

### Source Study

The derivation of the source studies included in the present meta-analysis was shown in **Figure S1**. A total of 9 relevant studies with IL-10, TNF-αgene polymorphisms and susceptibility to pSS were left for final inclusion in the meta-analyses. 7 articles were identified to assess the SNPs within IL-10 gene and pSS [17], [18], [19], [20], [21], [22], one of which also contained the data about TNF-α polymorphisms [23]. Then, the remaining 2 studies focused solely on TNF-α gene polymorphisms [24], [25]. All these 9 articles have been published in English in peer-review journals.

### Study Characteristic

The characteristics of each study were described in **Table 1** and **Table 2**. A collective total of 676 pSS patients and 1141 controls were included across the 7 studies related to IL-10 gene polymorphisms. These 7 studies were conducted in population samples from 6 Caucasians background and 1 Asians background. And sample sizes of these studies ranged from 147 to 462. Three studies utilized the American-European Consensus Group criteria to confirm a positive diagnosis of pSS, whereas two studies relied on the the Californian Criteria and European community Study Group Criteria for pSS, respectively. Apart from these 5 studies, the other two studies did not state which Criteria was employed (**Table 1**). As for TNF-α polymorphisms, there were only 3 studies including 723 cases and 1058 controls. All these 3 investigations were conducted in Caucasian groups consisted of 668 female and 54 male pSS patients, which fulfilled the American-European Consensus Group criteria. Hardy-Weinberg equilibrium test indicated that deviation from HWE could been observed in the genotype distribution in the included studies (**Table S1**).

**Table 1:** Characteristic of the 7 studies about the association between IL-10 polymorphisms and risk of pSS included in the meta-analysis.

Author	Year	Country	Ethnicity	Case/Control	Age	Women/Men┬	Criteria	Genotyping Method	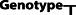	Genotype frequency		Alleles or Haplotypes Frequency		
										pSS	Control		pSS	Control
Hulkkonen	2001	Finland	Caucasian	62/400	60±11	60/2	Californian	PCR-RFLP	GCC/GCC	16	77	GCC	0.524	0.429
J, et al							Criteria		GCC/ACC	15	126			
									GCC/ATA	18	63	ACC	0.258	0.351
									ACC/ACC	5	46			
									ACC/ATA	7	63	ATA	0.218	0.22
									ATA/ATA	1	25			
Font	2002	Spain	Caucasian	63/150	57**┬**	59/4	European	PCR-RFLP	GCC/GCC	13	21	GCC	0.484	0.343
J, et al					(20–83)		community		GCC/ACC	17	45			
							diagnostic		GCC/ATA	18	16	ACC	0.246	0.387
							Criteria		ACC/ACC	2	18			
							In 1996		ACC/ATA	10	35	ATA	0.27	0.27
									ATA/ATA	3	15			
Origuchi	2003	Japan	Asian	47/107	NS	NS	NS	NS	GCC/GCC	0	0	GCC	0.085	0.065
T, et al									GCC/ACC	1	4			
									GCC/ATA	7	10	ACC	0.181	0.285
									ACC/ACC	1	6			
									ACC/ATA	14	45	ATA	0.734	0.65
									ATA/ATA	24	42			
Gottenberg	2003	France	Caucasian	129/96	56±13	115/14	America -	PCR-RFLP	GCC/GCC	27	20	GCC	0.442	0.396
JE, et al							European		GCC/ACC	30	15			
							Consensus		GCC/ATA	30	21	ACC	0.31	0.302
							criteria		ACC/ACC	13	13			
									ACC/ATA	24	17	ATA	0.248	0.302
									ATA/ATA	5	10			
Marka	2005	USA	Caucasian	99/135	55.6±11.8	97/2	America -	PCR-RFLP	−1082 A/G			−1082 A/G		
M, et al							European		AA	33	38	A	0.601	0.5741
							Consensus		AG	53	79			
							criteria		GG	13	18	G	0.399	0.4259
Willeke	2008	Germany	Caucasian	111/145	54.9±14.1	101/10	America -	PCR-RFLP	–	–	–	−1082 A/G		
P, et al							European		–	–	–	A	0.496	0.516
							Consensus		–	–	–	G	0.504	0.484
							criteria		–	–	–	−819 C/T		
									–	–	–	C	0.774	0.789
									–	–	–	T	0.226	0.211
									–	–	–	−592 A/G		
									–	–	–	A	0.774	0.789
									–	–	–	G	0.226	0.211
Limaye	2000	Australia	Caucasian	165/108	NS	NS	NS	NS	–	–	–	GCC	0.476	0.49
V, et al									–	–	–	ACC	0.322	0.286
									–	–	–	ATA	0.203	0.224

NS: None Stated.

┬:only the number of female and male pSS patients in these studies.

┬ “GCC”, “ACC”, “ATA” indicated G/A at −1082, C/T at −819, and C/A at −592 of the IL-10 gene.

? Mean year of pSS patients is 57, ranged 20–83 year in this study.

**Table 2:** Characteristics of articles included in the meta-analysis of TNF-α –308 polymorphisms and susceptibility to pSS.

Author	Country Year	Ethnicity	Case/Control	Age	Women/Men┬	Criteria	Genotyping Method	Genotype	Genotype frequency		“A” allele frequency	
									pSS	Control	pSS	Control
Correa PA, et al	Colombia 2005	Caucasian	67/430	49±13	67/0	America - European Consensus criteria	PCR	AAAGGG	13432	587338	0.27	0.11
Gottenberg JE, et al	France 2003	Caucasian	129/96	56±13	115/14	America - European Consensus criteria	PCR	AAAGGG	95070	31677	0.26	0.11
Bolstad AL, et al	Norway 2012/Sweden	Caucasian	527/532	57.9±13.2	487/40	American - European Consensus Criteria	Genotyping Assay	AAAGGG	–	–	0.34	0.20

┬:only the number of female and male pSS patients in these studies.

### TNF-α-308 A/G and Susceptibility to pSS

**Figure 1:**
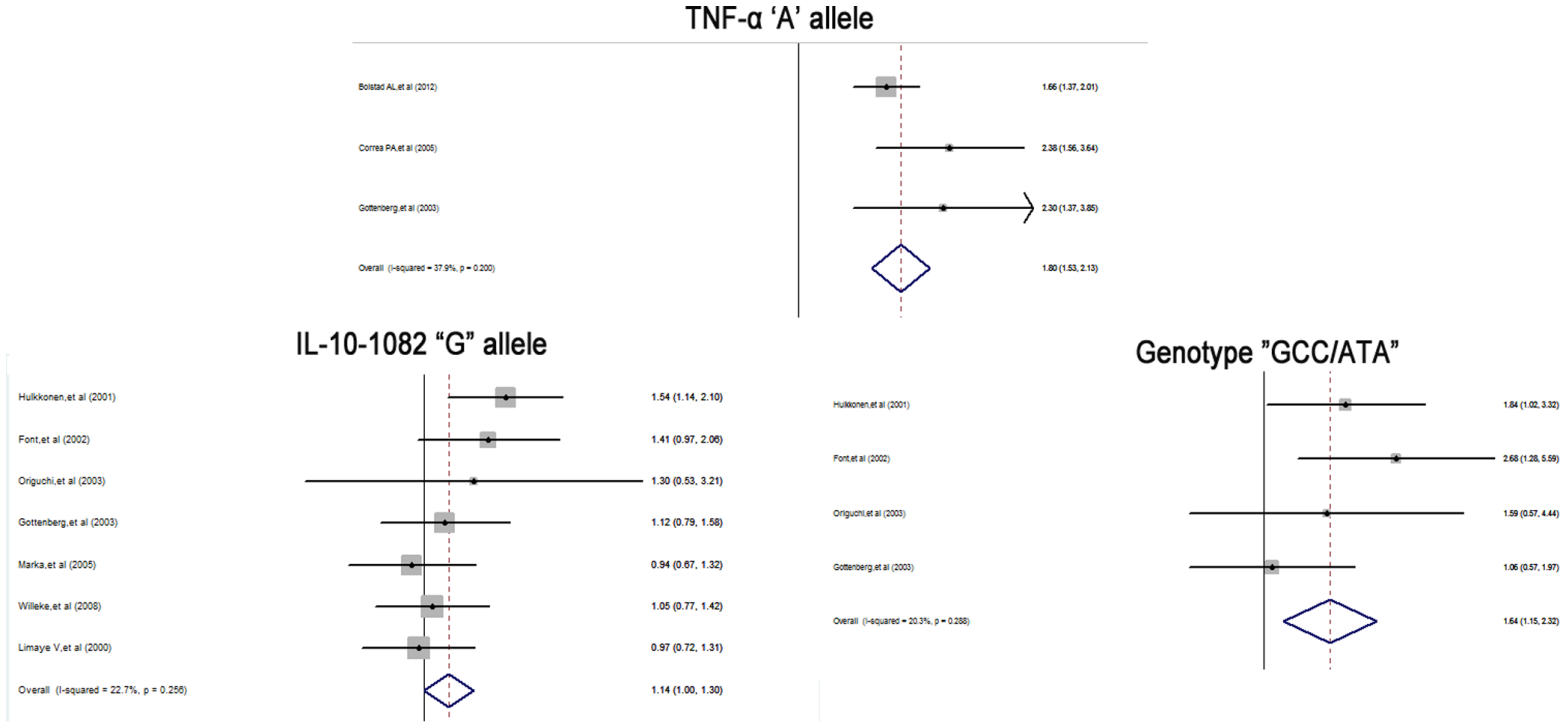
Forest plots for the association between TNF-α-308 “A” allele, IL-10-1082 “G” allele, genotype “GCC/ATA” and pSS risk in the overall population. (Meta-analyses with the fixed-effects model).

As mentioned above, there were 3 articles included in the present meta-analysis. All 3 studies have examined the influence of TNF-α-308 A/G on pSS patients, while only one study identified the role of TNF-α-238 A/G in pSS patients. A summary of the meta-analysis for TNF-α-308 A/G polymorphism and the risk of pSS was presented in **Figure 1**. The overall OR and 95% CI showed that TNF-α-308 A allele was significantly associated with pSS(OR = 1.8, 95%CI: 1.53–2.13, p<0.05). Heterogeneity assessment for TNF-α-308 A/G demonstrated that no significant inter-study variation existed in the meta-analysis(p = 0.24, I^2^ = 29.9%). Due to limited data, the meta-analyses for additive, dominant or recessive model of inheritance would not been performed. Besides, subgroup meta-analyses could not been carried out after stratification by ethnicity, HWE, gender, and other characteristics.

### IL-10-1082 A/G, −819T/C, −592A/C and Susceptibility to pSS

The additive, dominant and recessive models of inheritance were used to assess the association between reported IL-10-1082 A/G, -819T/C, -592A/C and susceptibility to pSS. Overall ORs for the associations demonstrated that no significant association of all these three polymorphisms under any model. Heterogeneity analyses revealed significant inter-study variation under additive and dominant models, particularly for -1082A/G. Conversely, no heterogeneity was observed in meta-analysis under the recessive model (**Table 3**). However, findings from the meta-analysis for the minor alleles of −1082A/G, −819T/C, −592A/C showed that the IL-10 −1082 G allele was a candidate genetic risk factor for pSS. And the genotype “GCC/ATA” was also determined to confer significant risk for pSS (**Figure 1**). Heterogeneity assessments showed that there was no significant heterogeneity among the studies with respect to the associations between these alleles, genotypes and pSS risk (**Table S2**).

**Table 3:** Meta-analysis of associations between IL-10 polymorphisms and pSS using the additive, dominant, recessive Model.

SNP	Genotype	Additive Model					Dominant Model					Recessive Model				
		OR with 95%CI	Q test	I^2^	Begg’s test	Egger’stest	OR with 95%CI	Q test	I^2^	Begg’s test	Egger’s test	OR with 95%CI	Q test	I^2^	Begg’s test	Egger’s test
−1082	GG															
	AG	1.13 (0.91,1.41)	0.044	59.2%	1	0.714	1.16 (0.99,1.34)	0.048	58.40%	1	0.799	0.88 (0.73,1.07)	0	84.6%	0.089	0.057
	AxA	1.47 (0.99,2.18)	0.025	68%	0.308	0.278										
−819	CC															
	TC	1.12 (0.96,1.31)	0.704	0	0.308	0.58	1.09 (0.94,1.25)	0.002	80.10%	0.734	0.227	1.04 (0.92,1.18)	0.719	0	0.734	0.88
	TT	0.57 (0.18,1.89)	0	84.5%	0.734	0.041										
−592	CC															
	AC	1.12 (0.96,1.31)	0.704	0	0.308	0.58	1.09 (0.94,1.25)	0.002	80.10%	0.734	0.227	1.04 (0.92,1.18)	0.719	0	0.734	0.88
	AA	0.57 (0.18,1.89)	0	84.5%	0.734	0.041										

### Haplotype and Risk of pSS

Based on the allele usage at the −1082, −819, −592 polymorphisms, the putative haplotypes were GCC, ACC, ATA within the promoter region between −1120 and −533 of IL-10 gene. The overall ORs suggested that the GCC carrier rate was significant higher in pSS patients than controls (OR = 1.18, 95%CI: 1.02–1.37), whereas GCC frequency did not differ between two groups (**Table 4**). The results suggested that GCC carriers may have an increased risk of pSS compared with these individuals with ACC, ATA haplotypes.

**Table 4:** Meta-analysis of association between Haplotypes (GCC, ACC, ATA) and pSS risk.

Haplotype	Haplotype carrier rate					Haplotype Frequency				
	OR with 95%CI	Heterogeneity		Publication bias		OR with 95%CI	Heterogeneity		Publication bias	
		Q test	I2 test	Begg’stest	Egger’stest		Q test	I2 test	Begg’s test	Egger’s test
GCC	1.18 (1.02,1.37)	0.911	0	0.734	0.543	1.10 (0.98,1.23)	0.599	0	0.462	0.485
ACC	0.87 (0.73,1.04)	0.476	0	0.089	0.375	0.88 (0.76,1.02)	0.191	34.6	0.086	0.108
ATA	1.04 (0.89,1.22)	0.898	0	1	0.865	0.97 (0.85,1.12)	0.826	0	1	0.266

### Subgroup Analysis by Ethnicity

Only one study was performed in Asian population among all included studies, thus we conducted the subgroup analyses by ethnicity (Caucasian). The results from these analyses showed that genotype “GCC/ATA” and GCC carrier were also risk factors for pSS in Caucasian population, which was consistent with that of the overall population. The meta-analysis showed the significant associations of −819T/C, −592A/C existed under the dominant model (OR = 1.59,95%CI:1.09–1.23), suggesting that individuals with genotype (CC+TC) or (CC+AC) may have a 59% increased risk of pSS in Caucasians population. In addition, the genotype ATA/ATA may be a protective factor against pSS in Caucasians(OR = 0.40,95%CI:0.19–0.84). However, no association was observed between IL-10 −1082 G allele and pSS in Caucasian group. The results of Heterogeneity assessments were listed in **Table S3**, the significant heterogeneity was observed under additive, dominant, recessive model for −1082 G/A.

### Sensitivity Analysis

Sensitivity analysis was conducted to assess the degree that each individual study affected the overall OR by the leave-one-out method that repeating the meta-analysis sequentially after excluding one study. As stated above, the significant association between −1082 G allele and pSS risk disappeared after excluding Origuchi’s study, which may be caused by small sample size and different ethnicity. Other results were relatively constant and stable in present study.

### Publication Bias

The funnel plot for association between TNF-α, IL-10 polymorphism and pSS was drawn, which did not show publication bias visually. Besides, both Begg’s test and Egger’s test were used to determine publication bias statistically. As expected, no publication bias was observed (Table 3, Table 4, Table S2, Table S3).

### Study Quality

According to NOS scoring system, the results of quality assessment demonstrated that 3 studies scored 7 stars, 2 studies scored 6 stars, 2 studies scored 5 stars, 1 study scored 4 stars and 1 study scored 3 stars.

## Discussion

The important role of genetic factors in the development of pSS was supported by the increased risk of pSS in first-degree relatives and siblings of pSS patients. The associations between SNPs of the TNF-α, IL-10 promoter region and susceptibility to pSS remained poorly elucidated owing to the conflicting data generated by independent studies. In present study, we conducted the meta-analyses of available studies to clarify these associations, confirming significant association between the minor allele of TNF-α-308 and pSS risk in the overall population. The meta-analyses also demonstrated that the minor allele “G” of IL-10 −1082 and genotype “GCC/ATA” contribute to the risk of pSS in the overall population. Besides, the OR generated by meta-analyses for haplotypes reflected that “GCC” carrier rate was relatively higher in pSS patients than controls.

In several previous studies, SNPs at positions −308 or −238 of TNF-α gene promoter region have been verified to be capable of elevating TNF-αexpression. There was considerable evidence supporting that the minor “A” allele of TNF-α-308 could contribute the improvement of TNF-αlevel as a potent transcriptional activator, leading to increased susceptibility of inflammation and autoimmunity [26], [27]. But the conflicting data from a range of independent studies would not support a risk role for TNF-308G/A in autoimmune diseases. Recently, a systematic Review and meta-analysis has also showed lack of association between TNF-α-308 and Primary biliary cirrhosis (PBC). However, our meta-analysis reflected that the minor “A” allele of TNF-308 conferred risk of pSS. In addition, Gottenbery’s study identified independently the positive association between TNF-308G/A and pSS patients with anti-SSB only. In this study, the linkage disequilibrium between the minor allele “A” and DRB1*03 has also been confirmed, which could affect anti-SSB antibody secretion [28].

IL-10 is an important immune-regulatory cytokine with diverse and intricate biological functions, which focused on suppression of TH1 cell, production of immunoglobulin and autoantibody synthesis [29]. It has been proposed that IL-10 expression was under strong genetic influences, and IL-10 locus may determine the contribution to genetic background for development of autoimmune disease [30]. However, the role of SNPs at −1082, −819, −592 of IL-10 gene as predisposition factors for pSS remained controversial. Our finding supported previous observations showing a significant association between the minor allele of IL-10 −1082 and susceptibility to pSS, but this phenomenon was not observed in population of Caucasian patients with pSS.

In the present study, a higher GCC haplotype carrier rate was found in pSS patients, and the genotype “GCC/ATA” was also significantly different between pSS patients and health controls. Our result was consistent with the finding of *Hulkonnen et al*, and *Font et al* that GCC frequency and GCC carrier rate were significantly higher in pSS patients than controls, suggesting GCC haplotype may be a predisposing factor for pSS. But no significant difference in GCC frequency was reported in *Limaye’s* and *Gottenberg’s* studies. In previous study, pSS patients who carried the “GCC” haplotype were identified to have a significantly elevated level of plasma IL-10 compared with GCC-negative patients [18]. The patients with GCC haplotype were also confirmed to have an earlier onset of pSS, whereas other investigators could not find the kind of difference [19], [21]. In other study, GCC haplotype was shown to be associated with clinical manifestation of some autoimmune disease. For example, the increased frequency of GCC haplotype was described in the SLE patients possessing Ro autoantibodies or renal involvement [31]. However, we could not conduct the meta-analysis about the role of SNPs or haplotypes within IL-10 gene in production of IL-10 or progression of disease in pSS patients due to the limited data. Although our analysis did not show significant associations between “ACC” frequency or carrier rate and pSS patients, the previous study has found that the presence of “AAC” haplotype was an independent factor associated with IgA anti-α-fodrin antibodies in pSS patients [22]. However, no significant associations of these SNPs, haplotypes with anti-SSA/SSB or gamma-globulins was detected. Except for “GCC” haplotype and −1082G alleles, the “GCC/ATA” genotype was also confirmed as an important component of genetic background in the susceptibility to pSS through our meta-analysis. The result showed that individuals who carried “GCC/ATA” genotype may have a 51% increased risk of pSS compared with other genotypes (OR = 1.51, 95%CI:1.14–2.00).

In the subgroup analyses by ethnicity, the −1082G allele was found to be insignificantly associated with pSS risk in Caucasian group (OR = 1.09, 95%CI:0.99–1.12). The significant association of −1082 G allele and pSS could be ruled out after excluding one Asian study, which indicated the overall OR was not stable and powerful. There was no sufficient power to detect a strong effect for −1082G allele on susceptibility to pSS due to small studies size. When all the studies were pooled into meta-analysis by Caucasians, there was also a significant association between genotype “ATA/ATA” and pSS risk, suggesting that the genotype “ATA/ATA” was a protective factor against pSS. In the previous study, patients with “ATA/ATA” genotype were confirmed to have lower IL-10 production in whole blood cultures than other genotypes [32]. Compared with GCC haplotype, ATA haplotype has been shown to have capacity to reduce IL-10 production by down-regulating the transcriptional activity. However, we did not observe any difference in ATA carriers rate or ATA frequency between pSS and controls, whatever in the overall population or Caucasian patients. With the exception of genotype “ATA/ATA”, we also found the significant association between −819T/C, −592 A/C and pSS risk under dominant model in Caucasian patients.

In recent, researchers have expanded our understanding of the genetic architecture of pathogenesis in several disease such as diabetes, SLE, MS, PBC through genome-wide association study (GWAS). GWAS has become a standard or novel method for disease gene discovery. In the previous studies, the researchers have demonstrated that STAT4 was a confirmed genetic risk factor for Sjögren’s syndrome [33]. HLA-DQA1*0501, HLA-DQB1*0201 and HLA-DRB1*0301 alleles were also identified to be risk factors for pSS, whereas HLA-DQA1*0201, HLA-DQA1*0301 and HLA-DQB1*0501 were possible protective factors [34]. However, the small sample size would affect the accuracy of these finding. Therefore, GWAS need to be conducted in a large cohort of pSS patients to further determine the role of these candidate genes in pSS. The application of GWAS approaches would identify several novel risk loci in pSS and provide clues to pathogenesis or new therapy targets of pSS [35].

It should be noted that the present study had some limitation which must be considered. Firstly, the number of available articles that fulfilled the selection criteria was only 9, thus the meta-analyses were restricted to a small patient population. Although some studies have shown the associations of age, autoantibodies, clinical manifestation and TNF-α,IL-10 polymorphisms, we could not conduct adequate subgroup meta-analysis by these factors. Secondly, only 1 of analyzed studies was conducted in Asian population, thus we could not determine the role of TNF-α,IL-10 polymorphisms in Asian pSS patients through the present meta-analyses. Thirdly, the interaction of different susceptibility genes and environment factors leaded to the disease, but our study could not assess gene-gene and gene–environment interactions due the limited information of included studies. In view of these limitations, further studies should focus on the associations of gene polymorphisms and clinical or laboratory characteristic in a large cohort of pSS patients.

In conclusion, our study confirmed that the minor allele “G” at position −308 of TNF-α gene was an independent risk factor for pSS. Of the three polymorphisms located in IL-10 promoter gene, IL-10-1082G allele and genotype “GCC/ATA” were found to be significantly associated with the disease. The haplotype GCC carrier rate was significant higher in pSS patients than controls. Future studies should be conducted in a large cohort of pSS patients to validate our findings. Besides, further investigations are also required to focus on the clinical relevance of these findings.

## Supporting Information

Figure S1 **Flowchart showing articles identification, inclusion and exclusion.**
(TIF)

Tables S1 **The HWE test for IL-1082, -819, -592 genotype distribution in included studies.**
(DOCX)

Tables S2 **Meta-analysis of association between IL-10 -1082 G, -819 T, -592 T, Genotype and pSS risk.**
(DOCX)

Tables S3 **Meta-analysis of associations between IL-10 polymorphisms and pSS risk in Caucasian population.**
(DOCX)
