# Supplementary figures and images for: The Association between TNF-α, IL-10 Gene Polymorphisms and Primary Sjögren’s Syndrome: A Meta-Analysis and Systemic Review
Source: PLoS One. 2013 May 21;8(5):e63401. doi: 10.1371/journal.pone.0063401 (PMC3661073; doi:10.1371/journal.pone.0063401)

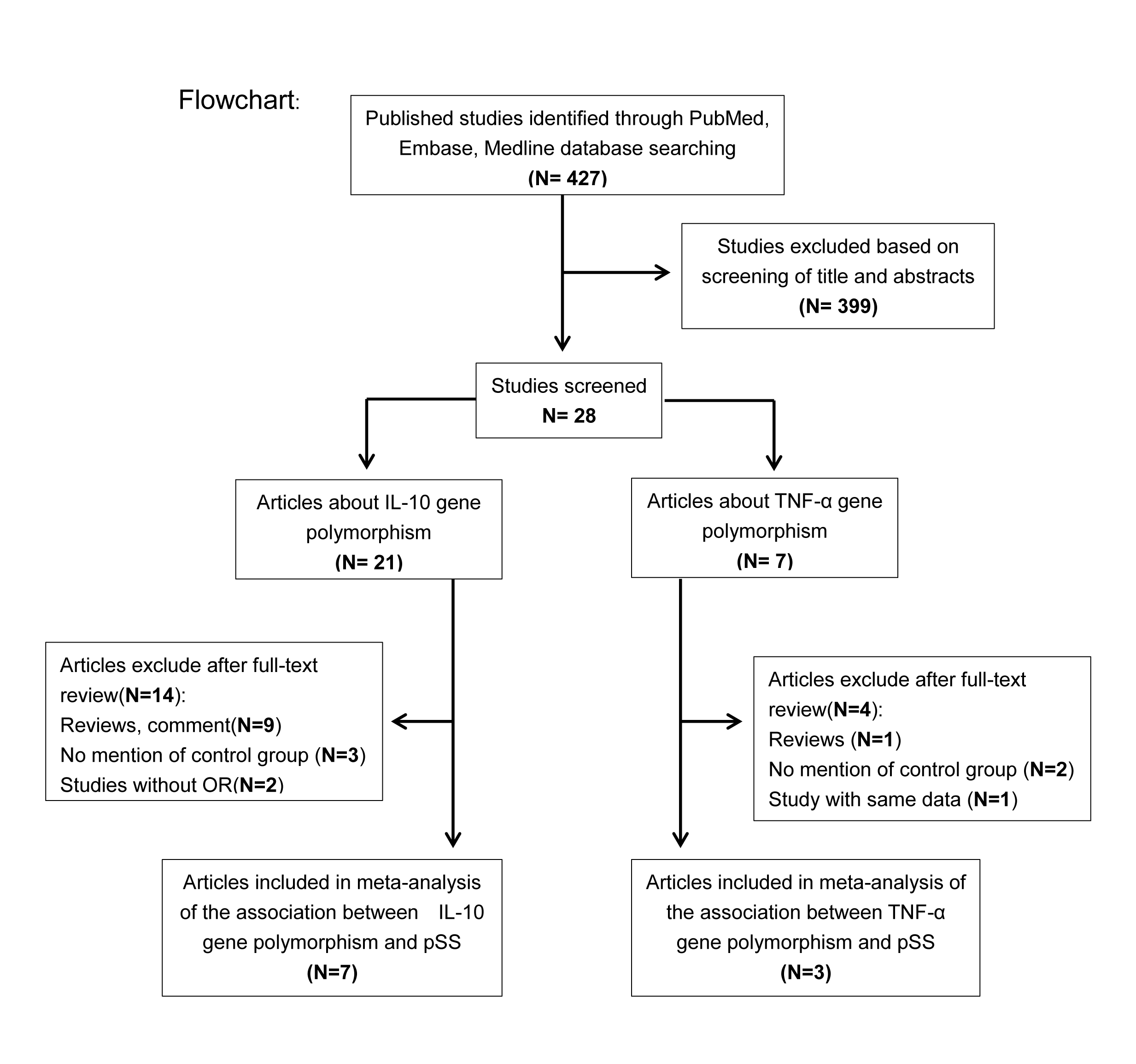

Supplement: Figure S1 — Flowchart showing articles identification, inclusion and exclusion. (TIF) [file pone.0063401.s001.tif]
