# Supplementary material for: The Association between TNF-α, IL-10 Gene Polymorphisms and Primary Sjögren’s Syndrome: A Meta-Analysis and Systemic Review
Source: PLoS One. 2013 May 21;8(5):e63401. doi: 10.1371/journal.pone.0063401 (PMC3661073; doi:10.1371/journal.pone.0063401)
Supplement: Tables S1 — The HWE test for IL-1082, -819, -592 genotype distribution in included studies. (DOCX) [file pone.0063401.s002.docx]

Table S1. The HWE test for IL-1082, -819, -592 genotype distribution in included studies.

| Study | -1082 | | -819 | | -592 | |
| --- | --- | --- | --- | --- | --- | --- |
|  | pSS | Control | pSS | Control | pSS | Control |
| Hulkkonen, et al | <0.05 | 0.479 | 0.148 | 0.1 | 0.148 | 0.1 |
| Font, et al | 0.373 | 0.229 | 0.31 | 0.092 | 0.31 | 0.092 |
| Origuchi, et al | 0.524 | 0.469 | 0.322 | 0.182 | 0.322 | 0.182 |
| Gottenberg, et al | 0.517 | 0.034 | 0.166 | 0.548 | 0.166 | 0.548 |
| Marka, et al | 0.247 | 0.022 | NS | NS | NS | NS |
| Willeke, et al | HWE^├^ | HWE | HWE | HWE | HWE | HWE |
| Limaye , et al | NS | NS | NS | NS | NS | NS |

NS: None Stated

├: No deviation from HWE was observed in this study and no sufficient data was used to calculate HWE test.
