# Supplementary material for: The Association between TNF-α, IL-10 Gene Polymorphisms and Primary Sjögren’s Syndrome: A Meta-Analysis and Systemic Review
Source: PLoS One. 2013 May 21;8(5):e63401. doi: 10.1371/journal.pone.0063401 (PMC3661073; doi:10.1371/journal.pone.0063401)
Supplement: Tables S2 — Meta-analysis of association between IL-10 -1082 G, -819 T, -592 T, Genotype and pSS risk. (DOCX) [file pone.0063401.s003.docx]

Table S2. Meta-analysis of association between IL-10 -1082 G, -819 T, -592 T, Genotype

and pSS risk

|  |  | OR with 95%CI | Heterogeneity | | Publication bias | |
| --- | --- | --- | --- | --- | --- | --- |
| Allele |  |  | Q test | I2 test | Begg's test | Egger's test |
| -1082 | minor Allele | 1.09(1.00,1.20) | 0.23 | 26% | 0.548 | 0.872 |
| -819 | minor Allele | 0.99(0.87,1.12) | 0.896 | 0 | 1 | 0.318 |
| -592 | minor Allele | 0.99(0.87,1.12) | 0.896 | 0 | 1 | 0.318 |
|  |  |  |  |  |  |  |
| Genotype | GCC/GCC | 1.19(0.87,1.63) | 0.702 | 0 | 1 | 0.763 |
|  | GCC/ACC | 0.97(0.73, 1.30) | 0.503 | 0 | 0.734 | 0.853 |
|  | GCC/ATA | 1.51(1.14, 2.00) | 0.27 | 23.6 | 1 | 0.765 |
|  | ACC/ACC | 0.60(0.36, 0.98) | 0.619 | 0 | 0.308 | 0.159 |
|  | ACC/ATA | 0.82(0.61, 1.11) | 0.819 | 0 | 0.734 | 0.661 |
|  | ATA/ATA | 0.81(0.56,1.16) | 0.069 | 57.70% | 0.734 | 0.046 |
