# Supplementary material for: The Association between TNF-α, IL-10 Gene Polymorphisms and Primary Sjögren’s Syndrome: A Meta-Analysis and Systemic Review
Source: PLoS One. 2013 May 21;8(5):e63401. doi: 10.1371/journal.pone.0063401 (PMC3661073; doi:10.1371/journal.pone.0063401)
Supplement: Tables S3 — Meta-analysis of associations between IL-10 polymorphisms and pSS risk in Caucasian population. (DOCX) [file pone.0063401.s004.docx]

Table S3. Meta-analysis of associations between IL-10 polymorphisms and pSS risk

in Caucasian population.

| Allele |  | | OR with 95%CI | Heterogeneity | | Publication bias | |
| --- | --- | --- | --- | --- | --- | --- | --- |
|  |  | |  | Q test | I2 test | Begg' test | Egger' test |
| -1082 | minor Allele | | 1.09(0.99-1.12) | 0.157 | 37.40% | 0.707 | 0.848 |
|  | GG vs AA | | 1.47(0.99,2.18) | 0.025 | 68% | 0.308 | 0.278 |
|  | AG vs AA | | 1.12(0.88,1.43) | 0.022 | 68.90% | 1 | 0.749 |
|  | GG+AG vs AA | | 1.15(0.98,1.35) | 0.023 | 68.50% | 1 | 0.813 |
|  | AA+AG vs GG | | 0.88(0.73,1.07) | 0 | 84.60% | 0.089 | 0.057 |
| -819 | minor Allele | | 0.96(0.82,1.12) | 0.919 | 0 | 0.462 | 0.19 |
|  | TT vs CC | | 0.43(0.21,0.88) | 0.823 | 0.00% | 1 | 0.713 |
|  | TC vs CC | | 1.13(0.94,1.37) | 0.549 | 0.00% | 0.296 | 0.202 |
|  | CC+TC vs TT | | 1.59(1.09,1.23) | 0.949 | 0.00% | 1 | 0.195 |
|  | TT+TC vs CC | | 1.03(0.87,1.23) | 0.547 | 0.00% | 1 | 0.14 |
| -592 | minor Allele | | 0.96(0.82,1.12) | 0.919 | 0 | 0.462 | 0.19 |
|  | AA vs CC | | 0.43(0.21,0.88) | 0.823 | 0.00% | 1 | 0.713 |
|  | AC vs CC | | 1.13(0.94,1.37) | 0.549 | 0.00% | 0.296 | 0.202 |
|  | CC+AC vs AA | | 1.59(1.09,1.23) | 0.949 | 0.00% | 1 | 0.195 |
|  | AA+AC vs CC | | 1.03(0.87,1.23) | 0.547 | 0.00% | 1 | 0.14 |
| Genotype | GCC/GCC | | 1.19(0.87,1.63) | 0.702 | 0 | 1 | 0.763 |
|  | GCC/ACC | | 0.99(0.74,1.32) | 0.346 | 5.80% | 0.296 | 0.05 |
|  | GCC/ATA | | 1.51(1.12, 2.03) | 0.14 | 49.10% | 1 | 0.628 |
|  | ACC/ACC | | 0.62(0.37, 1.05) | 0.46 | 0 | 0.296 | 0.152 |
|  | ACC/ATA | | 0.84(0.58,1.22) | 0.654 | 0 | 1 | 0.382 |
|  | ATA/ATA | | 0.40(0.19,0.84) | 0.867 | 0 | 1 | 0.524 |
| Haplotype Carrier Rate | | |  |  |  |  |  |
|  | | GCC | 1.18(1.02,1.37) | 0.776 | 0 | 0.296 | 0.05 |
|  | | ACC | 0.89(0.74, 1.08) | 0.366 | 0 | 0.296 | 0.104 |
|  | | ATA | 1.02(0.84,1.24) | 0.8 | 0 | 0.296 | 0.013 |
| Haplotype Frequency | | |  |  |  |  |  |
|  | | GCC | 1.10(0.98,1.23) | 0.451 | 0 | 0.308 | 0.21 |
|  | | ACC | 0.91(0.78,1.06) | 0.177 | 39.2% | 0.089 | 0.016 |
|  | | ATA | 0.94(0.79,1.11) | 0.912 | 0 | 0.308 | 0.03 |
